# Assessing Health-Related Quality of Life of Patients with Pulmonary Embolism with the Heart QoL Questionnaire

**DOI:** 10.3390/medicina61030370

**Published:** 2025-02-20

**Authors:** Niki Gkena, Paraskevi Kirgou, Ioannis C. Lampropoulos, Evangelos C. Fradelos, Dimitrios Papagiannis, Zoe Daniil, Konstantinos I. Gourgoulianis, Foteini Malli

**Affiliations:** 1 Department of Nursing, University of Thessaly, 41500 Larissa, Greece; gkenaniki@gmail.com (N.G.); paraskevi.kirgou@gmail.com (P.K.); ilampropoulos@uth.gr (I.C.L.); 2 Respiratory Medicine Department, Faculty of Medicine, School of Health Sciences, University of Thessaly, Biopolis, 41100 Larissa, Greece; zdaniil@uth.gr (Z.D.); kgourg@med.uth.gr (K.I.G.); 3 Laboratory of Clinical Nursing, Department of Nursing, University of Thessaly, 41500 Larissa, Greece; efradelos@uth.gr; 4 Public Health & Vaccines Lab, Department of Nursing, University of Thessaly, 41500 Larissa, Greece; dpapajohn@uth.gr

**Keywords:** pulmonary embolism, quality of life, heart QoL questionnaire, PE follow-up, chronic thromboembolic disease

## Abstract

*Background and Objectives*: While the acute phase of pulmonary embolism (PE) is well studied, its long-term physical and mental consequences have received less attention. Here, we aim to evaluate health-related quality of life (HRQoL) during the follow-up of PE with the Heart QoL questionnaire and to assess its reliability and validity as a standardized tool. *Materials and Methods*: A prospective study was conducted at the PE Outpatient Clinic of the University Hospital of Larissa, enrolling 100 PE patients (63% male, mean age 56.97 ± 16.09 years). The internal consistency of the Heart QoL questionnaire was measured using Cronbach’s alpha. Correlations between Heart QoL and SF-36 subscales were examined. *Results*: We included 100 patients with PE (63% males, 56.97 ± 16.09 years). A total of 59% of the patients reported reduced functional capacity post-PE. Heart QoL demonstrated excellent reliability (Cronbach’s alpha = 0.947), with strong inter-item correlations (range: 0.337–0.949). Internal consistency coefficients for Heart QoL subscales were 0.558 (global), 0.606 (physical), and 0.871 (emotional). The inter-item correlation range from 0.337 to 0.949. Mean Heart QoL scores were significantly lower than the Greek norms (global: 2.15 ± 0.829, physical: 2.17 ± 0.86, emotional: 2.09 ± 1.03). Heart QoL subscales exhibit significant correlations, with most SF-36 subscales indicating strong convergent validity. In the multiple linear regression analysis, MRC, dyspnea, reduced functionality, and the presence of symptoms were independent predictors of Heart QoL global and physical score. The presence of bleeding complications and reduced functionality were independent predictors of Heart QoL emotional score. *Conclusions*: Heart QoL is a reliable and valid tool for assessing HRQoL in PE patients, offering an alternative to more time-consuming tools. Dyspnea, reduced functionality, and bleeding complications significantly impact long-term HRQoL, underscoring the need for structured, multidisciplinary follow-up care that integrates both physical and mental health support in order to optimize patient long-term outcomes, especially in those at risk for chronic thromboembolic consequences.

## 1. Introduction

Pulmonary embolism (PE) is a severe and potentially life-threatening condition that arises due to the obstruction of pulmonary arteries by thrombotic emboli [1]. Despite advances in diagnostic techniques and treatment strategies, PE remains a significant global health challenge, with a substantial impact on morbidity, mortality, and healthcare systems [2]. While the acute phase of PE is well-characterized, increasing attention is being paid to the long-term consequences experienced by survivors, including persistent symptoms, reduced physical functionality, and psychological distress [3,4]. These sequelae can profoundly affect patients’ health-related quality of life (HRQoL), emphasizing the need for comprehensive assessment tools and structured follow-up care.

The definition of HRQoL encompasses the patients’ self-reported impact of the disease and treatment and refers to the perception on physical, mental and social functioning, and wellbeing [5,6], while it serves as a critical metric for evaluating the overall impact of chronic health conditions [7,8]. For PE patients, HRQoL may be compromised by factors such as residual dyspnea, post-thrombotic syndrome, and complications from anticoagulation therapy, including bleeding events [3,9,10,11]. However, the available data about HRQoL are scarce since the burden of the disease on HRQoL has received minimal attention. Understanding the factors that affect the long-term HRQoL impairment is crucial for guiding patient-centered care, improving outcomes, and informing clinical decision-making. In addition, limited data exist regarding the most appropriate instrument for the assessment of HRQoL of PE patients.

Current clinical guidelines emphasize the importance of follow-up care in PE management, focusing on physical rehabilitation, prevention of recurrent events, and management of anticoagulation therapy [1]. However, the assessment of long-term HRQoL impairments in PE patients remains underexplored and psychological and mental health aspects are often overlooked in routine care. Despite emerging evidence linking physical and mental health, there is a lack of standardized approaches for evaluating HRQoL in this population and no consensus exists on the most appropriate instrument for its assessment [4,7,8]. This gap has contributed to the absence of HRQoL considerations in current PE guidelines, as limited data are available to inform evidence-based recommendations. Given the increasing recognition of HRQoL as a key patient-centered outcome, further research is needed to establish valid, disease-specific measures and integrate them into clinical practice.

The primary aim of our research was to evaluate the applicability and reliability of the Heart QoL questionnaire, a tool previously validated in various cardiovascular condition within a cohort of PE patients [12,13,14]. Given the lack of standardized HRQoL assessment in PE, we compared Heart QoL outcomes with those derived from the Short Form 36 (SF-36) to evaluate its consistency and validity [15,16]. Additionally, we explored the relationship between HRQoL scores and key clinical and demographic characteristics, aiming to identify independent predictors of HRQoL outcomes. Despite growing recognition of the long-term impact of PE, HRQoL and further psychological consequences of PE remain insufficiently studied, and there is a critical need for research to determine the optimal follow-up approach [2] This study seeks to address these gaps by providing evidence on HRQoL assessment tools and identifying factors that influence patient-reported outcomes, contributing to a more comprehensive understanding of post-PE care.

## 2. Materials and Methods

### 2.1. Study Population

The current study is a cohort observational study of 100 patients with a first or recurrent episode of acute PE with or without deep vein thrombosis (DVT). Patients were prospectively enrolled during their follow-up at the PE Outpatient Clinic of the Respiratory Medicine Department of the University Hospital of Larissa, Greece. All participants had confirmed PE by computed tomography pulmonary angiography (CTPA) or ventilation/perfusion scan (V/Q scan). Concurrent DVT was diagnosed with whole leg compression ultrasonography. The inclusion criterion was objectively confirmed PE diagnosed before the 1st of January 2022. Patients < 18 years, pregnancy and/or dementia were excluded. Written informed consent was obtained from all participants. All participants completed the Short form 36 (SF-36), the Heart Quality of Life (Heart QoL) questionnaire, the Fatigue Assessment Scale (FAS), and the Post-VTE Functional Status (PVFS) scale. We calculated Charlson comorbidity index in order to assess for 10-year survival for every patient [17]. We recorded a detailed list of patient’s demographics and clinical features from their medical history. The study protocol was approved by the University of Thessaly ethics committee (Protocol No. 489/11-03-2022).

### 2.2. Assessment of Health-Related Quality of Life

We assessed HRQoL with the Heart QoL questionnaire, edited by the European Association of Preventive Cardiology [12]. The Heart QoL questionnaire is a 14-item questionnaire consisting of a global scale (14 items) made up of physical (10 items) and emotional (4 items) subscales [13]. A score is generated for each of the three Heart QoL scales as the mean of the number of items with a response. The maximum possible score in any scale is 3 and the minimum is 0, with higher scores indicating better health [13]. The Heart QoL questionnaire is designed to allow clinicians and researchers to assess baseline HRQL, to make between-diagnosis comparisons of HRQL, and to evaluate change in HRQL in patients with angina, myocardial infraction, or heart failure [14]. Heart QoL is offered by European Society of Cardiology in Greek. To the best of our knowledge, Heart QoL has not been previously used in patients with PE, although it has been validated in numerous cardiovascular conditions [18].

One of the most popular instruments for assessing health-related (HR) QoL is the 36-Item Short Form Health Survey questionnaire (SF-36) [19]. The SF-36 is a generic measure of health status as opposed to one that targets a specific age, disease, or treatment group. It has proven useful in comparing specific populations, estimating the relative burden of different diseases, differentiating the health benefits produced by a wide range of treatments applied, and screening individual patients [20]. The SF-36 measures eight scales: physical functioning (PF), role physical (RP), bodily pain (BP), general health (GH), vitality (VT), social functioning (SF), role emotional (RE), and mental health (MH), while it evaluates the general health change (HC) [19]. Component analyses show that there are two distinct concepts measured by the SF-36: a physical dimension, represented by the physical component summary (PCS,) and a mental dimension, represented by the mental component summary (MCS) [21]. The mean score is 50 and is considered a normative value for all subscales. Higher scores indicate better health status. This questionnaire is validated in the Greek language [16].

FAS is a questionnaire that investigates and evaluates the ability of the patient to cope in different activities. It consists of ten questions, five of them related to fatigue and the remaining five related to mental fatigue. Τhe total score comes out by adding the score of each question. Score on the FAS can range from 10 to 50 [22]. FAS is validated in the Greek language [23].

PVFS is used to assess the functional impairment following a VTE event [24]. PVFS ranges from 0 to 4 with “0” indicating no limitations and “4” accounting for severe limitations. There’s an extra scale “D” indicating death after VTE [24]. There are numerous questions that list each patient to the corresponding scale. All aspects of PVFS concern daily activities and functionality after VTE [25,26,27].

### 2.3. Outcome Measures

The primary outcome was the assessment of HRQoL with the Heart QoL questionnaire and to examine its internal consistency reliability. Additionally, we aimed to compare its results with those of the SF-36 instrument in order to examine its validity. We sought to compare HRQoL of PE patients with the HRQoL of the Greek population norms as established in the validation of the Greek version of SF-36 in 2005 [16] and in the EUROASPIRE IV Study of the European Society of Cardiology in 2016 [19. Moreover, secondary outcomes included the identification of potential demographic and clinical characteristics that could be predictive HRQoL in PE.

### 2.4. Statistical Analysis

Frequencies and percentages were used to represent the categorical variables. Medians (interquartile range-IQR) or means with the corresponding standard deviations (SD) were used to represent continuous variables. All answers were entered manually into the corresponding databases by one researcher. One-sample Kolmogorov–Smirnov test with a significance level of 0.05 was used to determine the normal distribution. Correlations between SF-36 and Heart QoL results, as well as among results and patients’ demographics and clinical features, were calculated using bivariate Spearman correlation coefficients and Kruskal–Wallis Test. Comparison of both questionnaires’ components with the Greek population norms was conducted by one-sample Wilcoxon rank sum test. The results of the SF36 components were categorized as “worse health” when the value was ≤50 and “better health” when the value was >50 compared to average [28,29]. We compared our results with the corresponding Greek norms [16]. We performed multiple linear regression analysis with SF-36 subscales and Heart QoL subscales as the dependent variables. The coefficient of determination (R squared, R^2^) was used to estimate the percentage of effect explained by the model. Demographics and clinical features with a significance level below <0.05 were retained for the analysis and served as possible independent predictors. For the validation of HRQoL, we assessed the internal consistency of its scales with the use of the Cronbach’s alpha index. Test–retest reliability was performed with the use of intra-class correlation coefficients. The inter-item correlation, the item-total statistic, and the intraclass correlation coefficient used for internal consistency reliability analysis. The pair-wised Spearman correlation coefficients were used for convergent validity assessment. Briefly, the validity of the Heart QoL questionnaire was assessed with the Spearman’s correlation coefficient of the two HRQoL instruments (Heart QoL and SF-36). A *p*-value < 0.05 was considered statistically significant. There were no missing data in the analysis. To ensure the study was statistically powered, the minimum sample size was calculated. This calculation considered the total population size of the targeted region, a margin of error of 5, with a confidence level of 95%. This calculation indicated that a minimum sample size of 97 is required in order to achieve the desired level of confidence and precision (https://www.enterprise-development.org/measuring-results-the-dced-standard/sample-size-calculator/) (accessed on 11 March 2022). IBM SPSS Statistics for Windows, Version 29.0. Armonk, NY: IBM Corp was used for data analysis.

## 3. Results

### 3.1. Patients

We included 100 patients with PE (63% males) while 24% had coexisting DVT. Mean age (±SD) was 56.97 ± 16.09 years. The patients’ sociodemographic characteristics are summarized in Table 1. Briefly, 59% of the patients reported reduced functional capacity during assessment (compared to prior PE diagnosis), 61% reported symptoms during the assessment (mainly exertional dyspnea and fatigue), 52% had a Medical Research Council (MRC) Dyspnea Scale ≥ 1 and 53% a New York Heart Association (NYHA) Classification ≥ 2. The majority (70%) received oral anticoagulation therapy and 14% had hemorrhagic complications during the course of therapy. Most of the patients (59%) had above 90% possibility of 10-year survival according to Charlson Comorbidity Index (CCI ≥ 5). High risk PE was observed in 7% of patients. Of the patients included, 9% had positive family history for VTE and 12% had a previous VTE event.

### 3.2. Health-Related Quality of Life

#### 3.2.1. Heart QoL

Mean Heart QoL global score was 2.15 ± 0.829, Heart QoL physical score was 2.17 ± 0.86, and Heart QoL emotional score was 2.09 ± 1.03 (Table 2). Heart QoL global score was high in 47%, medium in 19%, and low in 34% of patients. Heart QoL physical score was high in 52%, medium in 20%, and low in 28%. Heart QoL emotional score was high in 52%, medium in 19%, and low in 29% of patients. Mean Heart QoL global, physical, and emotional score in PE patients was significantly lower from the corresponding values of Greek population (Table 2) providing support for the presence of impaired HRQoL in the PE population.

#### 3.2.2. SF-36

Mean SF-36 physical component was 44.59 ± 13.90 and SF-36 mental component was 43.59 ± 19.78 (Supplementary Table S1). Of all patients, 54% reported worse physical health and 42% worse mental health, compared with their health prior to PE diagnosis. Regarding the answers on “change in health” questions, 59% of patients declared a health change after PE, 32% of them reported a better quality of life compared to prior PE diagnosis, and 27% reported a worse quality of life. We then compared SF-36 subscales to the corresponding normative values of the Greek population. Among SF-36 components PF, RP, RE, MH, SF, and GH mean scores were statistically significantly lower than the corresponding values of the Greek population (Table 2).

#### 3.2.3. FAS and PVFS

Mean FAS total score was 25.49 ± 7.82 (Supplementary Table S1). The evaluation of fatigue assessment indicated that 33% of the patients exhibits a fatigue rate of 10–20 on the scale FAS, that suggests poor fatigue level. A total of 25% of all patients scored a rate above 30, indicating high fatigue level. Compared with the mean of the Greek population, our sample mean is not different from the Greek norms (Table 3). As far as PVFS is concerned, 31% belonged to Grade 1, 19% to Grade 2, 11% to Grade 3, and 12% to Grade 4, and the remaining were categorized as Grade 0.

#### 3.2.4. Bivariate Correlations

Table 3 presents the significant bivariate correlations of Heart QoL score with SF-36 subscales and FAS. We observed that Heart QoL physical score was positively significantly correlated with Heart QoL global and emotional score, SF-36 physical score, SF-36 mental score, PH, RP, RE, VT, MH, BP, and GH. Additionally, Heart QoL emotional score was positively significantly correlated with Heart QoL global and physical score and SF-36 mental score, RE, VT, MH, and SF. Heart QoL global score was positively significantly correlated with Heart QoL emotional score, Heart QoL physical score, SF-36 physical component, SF-36 mental component, PF, RP, RE, VT, MH, SF, and GH. SF-36 physical component was significantly correlated with all SF-36 subscales (Table 3). SF-36 mental component was significantly correlated with all SF-36 subscales except BP.

FAS total score was significantly negatively correlated with all Heart QoL dimensions (global, physical, emotional component). FAS was significantly negatively correlated with the SF-36 mental component, RE, VT, and MH (Table 3).

### 3.3. Correlation of Health-Related Quality of Life Questionnaires with Clinicodemographic Characteristics

Table 4 presents the correlations of the HRQoL instruments with demographics and clinical characteristics of the population studied. Age was negatively significantly correlated with Heart QoL global score, Heart QoL physical score, and SF-36 physical component. We observed gender differences; males demonstrated statistically significantly higher levels in both Heart QoL as well as SF-36 subscales (Table 4). Higher educational level and residing in an urban and rural area (vs. living in a semi-urban area) is associated with significantly higher mean values of SF-36 physical component (Table 4).

The presence of symptoms was significantly associated with improved HRQoL (as assessed with Heart QoL global, mental, and physical score) and FAS subscales (Table 4). In keeping with the aforementioned findings, the absence of dyspnea as measured with MRC scale was associated with significantly increased mean values of Heart QoL global and physical score, indicating improved HRQoL (Table 4). In the same context, limitations in physical activity as assessed with NYHA classification was significantly associated with higher FAS total and FAS mental score (Table 4). Moreover, self-reported reduced functionality was significantly associated with worse HRQoL as assessed with Heart QoL global, physical, and emotional score, SF36 physical score, and with all FAS dimensions. Importantly, we observed that bleeding complications were associated with significantly reduced levels of Heart QoL emotional scale (Table 4).

### 3.4. Predictors of Heart QoL

We performed multiple linear regression analysis to assess for predictors of Heart QoL and the results are summarized in Supplementary Table S2. In the multiple linear regression analysis, Heart QoL global score predictors were MRC (β = −0.233, *p* = 0.035 r^2^ = 0.142, F = 8.022), reduced functionality (β = −0.719, *p* < 0.001, r^2^ = 0.184, F = 22.05), and the presence of symptoms (β = 0.195, *p* < 0.001, r^2^ = 0.117, F = 12.945). Heart QoL physical score predictors were MRC (β = −0.299, *p* = 0.007, r^2^ = 0.210, F = 12.862), CCI (β = −0.176, *p* < 0.001, r^2^ = 0.149, F = 17.125), reduced functionality (β = −0.742, *p* < 0.001, r^2^ = 0.18, F = 21.527), and the presence of symptoms (β = 0.195, *p* < 0.001, r^2^ = 0.107, F = 11.754). Heart QoL emotional score predictors were bleeding (β = −0.632, *p* = 0.033, r^2^ = 0.046, F = 4.689) and reduced functionality (β = −0.662, *p* = 0.001, r^2^ = 0.1, F = 10.932).

### 3.5. Psychometric Properties

#### 3.5.1. Internal Consistency and Reliability of Heart QoL

To assess the reliability of the Heart QoL questionnaire, we calculated the Cronbach’s alpha value, the inter-item correlation, the item-total statistic, and the intra-class correlation coefficient. Cronbach’s alpha is considered acceptable when its value is above 0.7 [30] and in our sample the value was 0.947. We conducted an additional calculation for the physical and emotional subscales and the Cronbach’s alpha was 0.939 and 0.964, respectively. All dimensions associated statistically significant positive with each other according to inter-item correlation (range from 0.337 to 0.949). Item-total correlations scored from 0.620 to 0.808, indicating very good discrimination. Intra-class correlation coefficients were 0.558 for global, 0.606 for physical, indicating moderate reliability and 0.871 for emotional score, indicating good reliability.

#### 3.5.2. Validity

To assess construct validity, we used the pair-wised Spearman correlation coefficients between Heart QoL and SF-36 subscales and overall scores (convergent validity). Table 4 shows all correlations with general good validity; thus, the Heart QoL global score had significant correlation with all SF-36 subscales (except BP and HC), Heart QoL physical score did not correlate only with HC, and Heart QoL emotional score had correlation with RE, VT, MH, SF, and SF-36 MHS. Also, all Heart QoL scores had strong negative correlation with all FAS dimensions.

As far as the clinical characteristics are concerned, MRC and CCI had significant correlations with global and physical Heart QoL while reduced functionality correlated negatively with all Heart QoL subscales (Table 4). In general, Heart QoL questionnaire showed a good discriminant validity.

## 4. Discussion

This study addresses a critical gap in the literature by evaluating HRQoL in PE patients using the Heart QoL questionnaire. Heart QoL displayed high internal consistency, reliability, and a good discriminant validity. Our results suggest that Heart QoL, which has been used in several cardiovascular conditions, can be used in PE patients and since it consists of only 14 items, it may be more time efficient than other instruments of HRQoL previously studied in PE. Additionally, we observed impaired HRQoL of PE patients during their follow-up, which is independently associated with the presence of symptoms (mainly dyspnea), reduced functionality and bleeding complications. Our findings suggest that Heart QoL could be incorporated into routine clinical practice, facilitating a standardized and efficient HRQoL assessment for PE patients with a special interest in patients at risk of chronic thromboembolic disease.

We demonstrate significantly impaired HRQoL in PE patients, as indicated by both Heart QoL and SF-36. All Heart QoL subscales indicated low levels of HRQoL, aligning with SF-36 values, which were below the Greek normative data. Our findings are in harmony with previously published data [22,31,32,33,34] reinforcing that HRQoL remains compromised during the first year post-PE, particularly among patients with dyspnea, as reported by Valerio et al. [32] and Kahn et al. [34]. In the same concept, the prospective evaluation of HRQoL demonstrated impairments that improved over time [32]. Taking the above into account, our results provide further support to the need for chronic follow-up of PE, especially in patients at risk of chronic thromboembolic disease, and highlight the importance of structured post-PE care to improve long-term outcomes.

We were able to identify some significant independent predictors of HRQoL. The presence of symptoms, especially dyspnea, and reduced functionality, had a negative impact in HRQoL. Identifying high-risk subgroups who are more likely to develop mental health complications, may help optimize follow-up care and resources allocation. Early recognition may enable targeted interventions, such as psychological support. Importantly, the presence of bleeding independently predicted the emotional Heart QoL subscale. Previous studies in patients with VTE present similar results [35,36,37,38]. Researchers have identified obesity, active malignancy, and cardiopulmonary comorbidity as independent predictors of HRQoL post PE [22]. The discrepancy of the aforementioned findings with our study may be attributed to differences in sample size and population characteristics. The negative association of bleeding events with HRQoL observed in our study may not be limited to PE; bleeding associated with antithrombotic therapy is associated with higher anxiety levels and clinically relevant lower HRQoL scores, providing further support for its’ possible adverse impact in mental health [39]. These findings suggest that bleeding should not be viewed solely as a physical complication, but as a factor that influences mental wellbeing, reinforcing the need for shared decision-making process. A patient-centered approach that incorporates HRQoL concerns may be valuable for facilitating more substantive care and risk discussions, especially when it concerns the continuation of anticoagulation therapy [40,41].

A key contribution of our study is the validation of Heart QoL in patients with PE. We observed high internal consistency, reliability, with good inter-item and item-total correlations as confirmed by Cronbach’s alpha values. The significant correlations between the Heart QoL and SF-36 indicate strong convergent validity, while the significant correlations between Heart QoL and patient clinical characteristics support its’ discriminant validity. The aforementioned results, as well the significant associations of Heart QoL with other HRQoL instruments, suggest that Heart QoL is a suitable instrument for the evaluation of HRQoL in PE patients, offering an alternative to more comprehensive but time-consuming tools such as SF-36 and PEmbQoL [5,42,43,44,45,46], some of which have been previously validated in Greek [44]. An important advantage of Heart QoL is its concise structure consisting of only 14 items, making it less burdensome for patients while still providing a comprehensive evaluation of the mental health impact of PE.

PE is an acute disease; however, many patients may experience long-term complications. Our study adds to the growing evidence that psychological distress is a major, yet underrecognized, consequence of PE. Current guidelines focus on physical rehabilitation, anticoagulation management, prevention of recurrences, and investigation of chronic thromboembolic pulmonary hypertension, but they lack recommendations on HRQoL assessment and mental health care [4]. Our results and previously published data [44] support the inclusion of HRQoL as a standard measure in PE follow-up, helping clinicians to detect psychological distress and functional impairment that may require intervention.

Despite the strengths of our study, it has some limitations. We acknowledge that our sample size is relatively small. However, our study’s sample size was determined through statistical power calculations to ensure sufficient validity of the findings. Additionally, this is a single center study from one tertiary center. However, our PE clinic is the single one in a population of 718,640, while the demographic characteristics of our study suggest that we can extrapolate our results in a larger population. We acknowledge that further studies with larger and more diverse samples could enhance the external validity and broader applicability of the results. Furthermore, our study lacks longitudinal exploration, limiting our ability to assess long-term outcomes and causality. Future research incorporating longitudinal designs could provide deeper insights into the progression of the observed effects over time. Although we employed standardized data collection methods, there is always a possibility of minor inaccuracies in self-reported data, which could introduce measurement bias. Finally, due to sample size limitations, performing meaningful subgroup analyses was not feasible in our study.

## 5. Conclusions

In conclusion, our study provides valuable insights into HRQoL of PE patients, utilizing the Heart QoL questionnaire for the first time in this population. The results demonstrate that Heart QoL is a reliable, valid, and efficient tool for assessing HRQoL, offering practical advantages due to its brevity compared to other instruments. Importantly, our findings highlight the significant impairment of HRQoL in PE patients, which is influenced by persistent symptoms such as dyspnea, reduced functionality, and bleeding complications associated with treatment. These factors underscore the necessity of a structured, multidisciplinary follow-up that addresses both physical and mental health outcomes. Ultimately, integrating mental health support into PE management represents a crucial step forward in addressing the comprehensive needs of this patient population.

## Figures and Tables

**Table 1:** Sociodemographic and clinical characteristics of study population.

Characteristic	Mean ± SD or %
***Age*** (years) ***Gender*** (male/female) ***Smoking status*** (non-smoker/ex-smoker/current smoker) ***BMI*** (healthy weight/overweight/obesity) ***Educational level*** (primary school/3 years of high school/6 years of high school/university graduates/MSc and PhD graduates) ***Residence*** (urban/rural/semi-urban) ***Working status*** (unemployed/retired/employee/farmer) ***Marital status*** (married/in a relationship/single/widower) ***Comorbidities*** (none/≥1) ***MRC*** *(*0/≥1) ***NYHA*** *(0*/≥1) ***Anticoagulation therapy*** *(*yes/no) ***Bleeding*** *(*yes/no) ***Reduced Functionality*** *(*yes/no) ***Symptoms*** (yes/no) ***Charlson Comorbidity Index, CCI*** *(*0/1/2/3/4/5/6/7)	56.97 ± 16.09 63%/37% 47%/36%/17% 24%/35%/41% 29%/12%/31%/26%/2% 67%/26%/7% 7%/47%/41%/5% 63%/28%/9% 19%/81% 48%/52% 47%/53% 70%/30% 14%/86% 59%/41% 61%/39% 28%/14%/17%/17%/12%/6%/4%/2%

**Table 2:** Heart QoL, SF-36, FAS results compared to the Greek healthy population norms.

Parameters	Mean(±SD)	Mean (±SD) of the Greek Population
Global Heart QoL	2.15 (±0.82), *p* < 0.001	2.49 (±0.56)
Physical Heart QoL	2.17 (±0.86), *p* < 0.001	2.50 (±0.61)
Emotional Heart QoL	2.09 (±1.03), *p* < 0.001	2.48 (±0.68)
Physical functioning	69.65 (±31.6), *p* < 0.001	80.76 (±25.62)
Physical role functioning	63 (±43), *p* < 0.001	79.74 (±37.72)
Emotional role functioning	69.33 (±44.1), *p* < 0.001	81.53 (±36.31)
Vitality	64.2 (±27.7), *p* = 0.222	66.53 (±22.39)
Mental health	65.36 (±16.2), *p* = 0.003	68.23 (±21.26)
Social role functioning	75.87 (±32.53), *p* < 0.001	82.05 (±28.12)
Bodily pain	75.25 (±31.88), *p* = 0.303	72.98 (±31.66)
General health perceptions	56.15 (±29.63), *p* < 0.001	67.46 (±23.54)
Total FAS	25.49 (±7.82), *p* = 0.1	26.46 (±6.39)
Physical FAS	14.81 (±3.25)	-
Mental FAS	10.68 (±5.53)	-

**Table 3:** Bivariate correlations between the Heart QoL, SF-36, and FAS scores. Abbreviations: PF, physical functioning; RP, physical role functioning;.

Measure	Compared Parameter	*p*-Value	Correlation (r)
Heart QoL global	Heart QoL physical	<0.001	0.952
	Heart QoL emotional	<0.001	0.780
	SF-36 PF	0.003	0.297
	SF-36 RP	0.011	0.255
	SF-36 RE	<0.001	0.354
	SF-36 VT	<0.001	0.435
	SF-36 MH	0.002	0.310
	SF-36 SF	0.023	0.228
	SF-36 GH	0.013	0.247
	SF-36 PCS	0.046	0.200
	SF-36 MCS	<0.001	0.363
	FAS Total	<0.001	−0.796
	FAS physical	<0.001	−0.736
	FAS mental	<0.001	−0.697
Heart QoL physical	Heart QoL emotional	<0.001	0.583
	SF-36 PF	0.001	0.315
	SF-36 RP	0.009	0.260
	SF-36 RE	0.012	0.250
	SF-36 VT	<0.001	0.381
	SF-36 MH	0.005	0.280
	SF-36 BP	0.029	0.218
	SF-36 GH	0.013	0.249
	SF-36 PCS	0.010	0.257
	SF-36 MCS	0.007	0.269
	FAS total	<0.001	−0.725
	FAS physical	<0.001	−0.739
	FAS mental	<0.001	−0.602
Heart QoL emotional	SF-36 RE	<0.001	0.435
	SF-36 VT	<0.001	0.383
	SF-36 MH	0.003	0.297
	SF-36 SF	0.036	0.210
	SF-36 MCS	<0.001	0.455
	FAS total	<0.001	−0.691
	FAS physical	<0.001	−0.492
	FAS mental	<0.001	−0.684
FAS total	FAS physical	<0.001	0.766
	FAS mental	<0.001	0.939
FAS physical	FAS mental	<0.001	0.537

RE, emotional role functioning; VT, vitality; MH, mental health; SF, social role functioning; BP, bodily.

**Table 4:** Significant differences of Heart QoL, SF-36, and FAS values according to clinicodemographic characteristics. Abbreviations: SD, standard deviation.

Measure	Characteristic	Mean (SD)	*p*-Value
Heart QoL Global	Gender (male vs. female)	2.3 (0.73) vs. 1.90 (0.92)	0.029
	MRC (0 vs. ≥1)	2.36 (0.77) vs. 1.37 (0.86)	0.011
	Reduced functionality (no vs. yes)	2.58 (0.44) vs. 1.86 (0.90)	<0.001
	Symptoms (cough vs. fatigue vs. dyspnea vs. weakness vs. exertional dyspnea vs. none)	1.62 (1.18) vs. 1.97 (0.71) vs. 1.65 (0.98) vs. 1.71 (1.23) vs. 2.33 (0.47) vs. 2.58 (0.53)	<0.001
Heart QoL Physical	Gender (male vs. female)	2.33 (0.77) vs. 1.91 (0.96)	0.027
	MRC (0 vs. ≥1)	2.41 (0.78) vs. 1.39 (0.60)	0.005
	CCI (0 vs. 1 vs. 2 vs. 3 vs. 4 vs. 5 vs. 6 vs. 7)	2.55 (0.55) vs. 2.5 (0.53) vs. 2.12 (0.9) vs. 1.84 (1.06) vs. 2.08 (0.8) vs. 1.91(0.84) vs. 1.1 (1.32) vs. 1.45 (1.2)	0.032
	Symptoms (cough vs. fatigue vs. dyspnea vs. weakness vs. exertional dyspnea vs. none)	1.83 (1.09) vs. 1.98 (0.74) vs. 1.56 (1.04) vs. 1.78 (1.18) vs. 2.23 (0.49) vs. 2.63 (0.61)	<0.001
	Reduced functionality (no vs. yes)	2.61 (0.49) vs. 1.87 (0.93)	<0.001
Heart QoL Emotional	Gender (male vs. female)	2.23 (0.98) vs. 1.86 (1.08)	0.044
	Bleeding (no vs. yes)	2.18 (0.98) vs. 1.55 (1.16)	0.047
	Reduced functionality (no vs. yes)	2.48 (0.61) vs. 1.82 (1.17)	0.008
SF-36 MCS	Gender (male vs. female)	47.13 (17.05) vs. 36.04 (22.09)	0.015
SF-36 PCS	Reduced functionality (no vs. yes)	47.92 (12.97) vs. 42.27 (14.17)	0.043
	Educational status (primary school vs. 3 years of high school vs. 6 years of high school vs. university graduates vs. MSc/PhD graduates)	37.09 (14.81) vs. 45.04 (12.67) vs. 48.38 (11.28) vs. 47.72 (13.95) vs. 50.89 (11.78)	0.009
	Residence (urban vs. rural vs. semi-urban)	45.94 (13.48) vs. 44.38 (14.25) vs. 32.41 (12.34)	0.043
	Working status (unemployed vs. retired vs. employee vs. farmer)	48.01 (9.34) vs. 39.11 (14.82) vs. 49.18 (10.66) vs. 52.68 (17.13)	0.014
FAS Total	Reduced functionality (no vs. yes)	22.09 (5.71) vs. 27.84 (8.25)	0.001
	Symptoms (cough vs. fatigue vs. dyspnea vs. weakness vs. exertional dyspnea vs. none)	34.66 (7.09) vs. 26.74 (6.69) vs. 29.3 (9.55) vs. 29.8 (9.57) vs. 21 (1.73) vs21.92 (6.44)	0.003
	NYHA (0 vs. ≥1)	24.87 (7.59) vs. 32.16 (5.87)	0.033
FAS Physical	Reduced functionality (no vs. yes)	13.36 (2.41) vs. 15.81 (3.4)	<0.001
	Symptoms (cough vs. fatigue vs. dyspnea vs. weakness vs. exertional dyspnea vs. none)	17.33 (2.3) vs. 15.54 (2.74) vs. 16.4 (3.74) vs. 15.3 (3.68) vs. 15 (10) vs. 13.41 (3.20)	0.006
FAS Mental	Reduced functionality (no vs. yes)	8.73 (4.44) vs. 12.03 (5.83)	0.006
	Symptoms (cough vs. fatigue vs. dyspnea vs. weakness vs. exertional dyspnea vs. none)	17.33 (5.5) vs. 11.2 (5.01) vs. 12.9 (6.55) vs. 14.5 (6.63) vs. 6.00 (1.00) vs. 8.51 (4.42)	0.006
	NYHA (0 vs. ≥1)	10.23 (5.38) vs. 15.66 (4.27)	0.038

## Data Availability

Data are available upon request.

## Supplementary Materials

The following supporting information can be downloaded at: https://www.mdpi.com/article/10.3390/medicina61030370/s1, Supplementary Table S1: Results of Heart QoL, SF-36 and FAS; Supplementary Table S2: Predictors of Heart QoL questionnaire.
