# Supplementary material for: Assessing Health-Related Quality of Life of Patients with Pulmonary Embolism with the Heart QoL Questionnaire
Source: Medicina (Kaunas). 2025 Feb 20;61(3):370. doi: 10.3390/medicina61030370 (PMC11944180; doi:10.3390/medicina61030370)
Supplement: Supplementary file 1 [file medicina-61-00370-s001.zip › medicina-3444462-supplementary.pdf]

Table S1. Results of Heart QoL, SF-36 and FAS.

	Mean	Standard Deviation	95%CIs
**Heart QoL**			
Global score	2.15	0.82	1.99-2.32
Physical score	2.17	0.86	2.00-2.35
Emotional score	2.09	1.03	1.89-2.30
**SF-36**			
Physical functioning	69.65	31.60	63.37-75.92
Role physical functioning	63.00	43.00	54.46-71.53
Emotional role	69.33	44.10	60.58-78.08
Vitality	64.20	27.70	58.70-69.69
Mental health	65.36	16.20	62.14-68.57
Social role functioning	75.87	32.53	69.42-82.32
Bodily pain	75.25	31.88	68.92-81.57
General health perceptions	56.15	29.63	50.26-62.03
Physical component	44.58	13.90	41.82-47.34
Mental component	43.59	19.78	39.12-46.94
**Fatigue Assessment Scale**			
Total score	25.49	7.82	23.93-27.04
Physical score	14.81	3.25	14.16-15.45
Mental score	10.68	5.53	9.58-11.78

Table S2. Predictors of Heart QoL questionnaire.

	Heart QoL global			Heart QoL physical			Heart QoL emotional		
	β	p	r^2	β	p	r^2	β	p	r^2
**Reduced functionality**	-0.719	<0.001	0.184	-0.742	<0.001	0.18	-0.662	0.001	0.1
**Symptoms**	0.195	<0.001	0.117	0.195	<0.001	0.107	-	-	-
**MRC**	-0.233	0.035	0.142	-0.299	0.007	0.210	-	-	-
**CCI**	-	-	-	-0.176	<0.001	0.149	-	-	-
**Bleeding**	-	-	-	-	-	-	-0.632	0.033	0.046
